# The Immunogenetics of Autoimmune Cholestasis

**DOI:** 10.1016/j.cld.2015.08.002

**Published:** 2016-02

**Authors:** Palak J. Trivedi, Gideon M. Hirschfield

**Affiliations:** National Institute of Health Research (NIHR) Birmingham Liver Biomedical Research Unit, Institute of Immunology and Immunotherapy, University of Birmingham, Wolfson Drive, Birmingham B15 2TT, UK

**Keywords:** Autoimmunity, Autoimmune liver disease, Mucosal immunity, Primary sclerosing cholangitis, Primary biliary cirrhosis

## Abstract

The immune-mediated hepatobiliary diseases, primary biliary cirrhosis and primary sclerosing cholangitis are relatively rare, albeit and account for a significant amount of liver transplant activity and liver-related mortality globally. Precise disease mechanisms are yet to be described although a contributory role of genetic predisposition is firmly established. In addition to links with the major histocompatibility complex, a number of associations outside this region harbor additional loci which underscore the fundamental role of breaks in immune tolerance and mucosal immunogenicity in the pathogenesis of autoimmune biliary disease. We provide an overview of these key discoveries before discussing putative avenues of therapeutic exploitation based on existing findings.

## Key points

• The strongest genetic associations in primary biliary cirrhosis (PBC) and primary sclerosing cholangitis (PSC) occupy distinct regions of the major histocompatibility complex (MHC).
• Most non-MHC associations overlap with other autoimmune diseases, with putative risk loci indicating altered immunoregulatory pathways, aberrant microbial handling and dysregulated mucosal immunity generally.
• Less than 20% of the expected heritability is explained by currently available genome-wide studies.
• Epigenetics have provided insight into sex predisposition as well as overexuberant chemokine-mediated lymphocyte recruitment in the pathogenesis of immune-mediated liver disease.
• Recognition of definitive immune regulatory mechanisms and pathway defects may facilitate approaches to risk stratification as well as in the identification of ostensible therapeutic avenues.

## Introduction

Chronic cholestatic liver diseases encompass a range of disorders affecting the hepatobiliary system and arise secondary to a variety of causes, including molecular defects caused by genetic variation or drugs, structural changes due to congenital disorders, or autoreactive bile duct injury.^1^ In clinical practice, the latter is most often applied in reference to primary biliary cirrhosis (PBC) and primary sclerosing cholangitis (PSC), themselves part of the broader spectrum of immune-mediated liver disease.^2^ Support of an autoimmune cause is provided by strong genetic links with human leukocyte antigen (HLA), the presence of high circulating autoantibody titers, and a clear increased frequency of concomitant autoimmune disease in affected individuals as well as associated family members.

However, unlike many classic autoimmune syndromes, PBC and PSC do not typically respond to immunosuppressive therapy; with the development of newer therapeutic interventions being significantly mired by gaps in understanding disease etiopathogenesis. Nevertheless, recent developments have begun to dissect the impact of certain genetic polymorphisms not only on predisposition but also varying phenotypic presentations, susceptibility to progressive disease, and putative therapeutic avenues based on the rational targeting of immune pathways presumed relevant to disease initiation.

Genetic exploration of rare diseases frequently establish major genes that regulate pathogen-specific immune responses, and genome-wide association studies (GWAS) have been increasingly productive for recognizing common variants within a given population. However, identifying the exact genes that result in statistical associations is often not possible to determine, and often many plausible candidates at a given susceptibility locus are proposed.^3^ Conversely, if only one candidate susceptibility gene is identified, the associated causative variant is often unknown.^4^

## Epidemiologic considerations: heritability and familial clustering

Although PBC and PSC represent relatively rare disease entities, systematic reviews of disease frequency suggest an increasing incidence and prevalence globally.^5^ Moreover, both conditions continue to pose a significant burden on health care services, accounting for approximately 25% of all first liver transplantations in the Western world.^6^ For PBC, clustering of cases has been reported in certain geographic areas, for instance, in coastal First Nations of British Columbia where disease prevalence is as high as 25% within generations of well-characterized multiplex families.^7^ Studies of monozygotic twins provide further support of a genetic predisposition, with a reported 63% concordance rate, among the highest reported for any autoimmune disease.^8^ Moreover, a family history seems to be one of the strongest identified risk factors for disease development (odds ratio: 10.7), with approximately 6% of the patients having an affected first-degree relative.^9^ Conversely, population studies from Australia estimate a prevalence of PBC between 19.1 per million among birth natives relative and 183 per million among those migrating to the continent from Europe.^10^, ^11^ Although these data support an inherent genetic predisposition to disease development, the incidence seems to decrease in consecutive generations of descendants of European migrants possibly indicating the impact of environmental influences.^12^

Heritable aspects of PSC are also evinced through family studies, wherein disease prevalence in first-degree relatives of affected patients is 100-times greater than that observed across unrelated comparator populations.^13^ Clinical associations between PSC and colonic inflammatory bowel disease (IBD) are well described,^14^ and the risk of developing PSC and/or ulcerative colitis (UC) is also significantly increased in families of afflicted individuals compared with controls.^15^

**Fig. 1:**
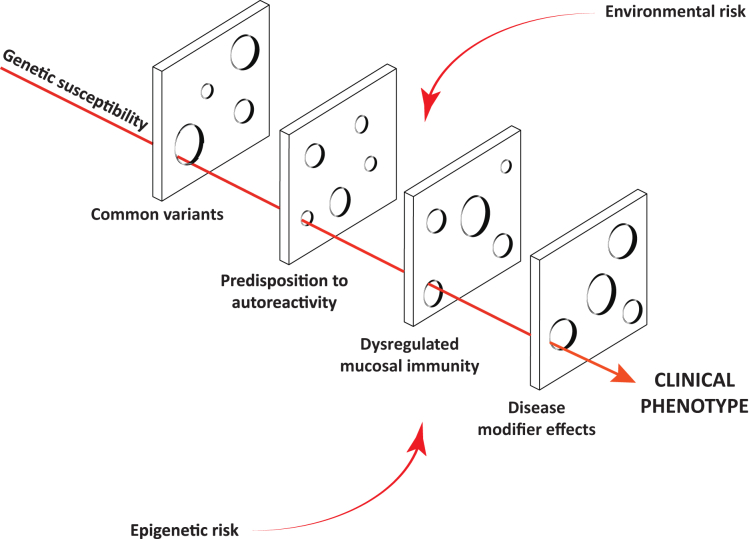
Aggregation of genetic risk in complex diseases. PBC and PSC represent complex diseases, in which the cause is attributed to presence of ostensible genetic risk factors that exhibit a poorly understood interaction with coexisting environmental influences. Individual susceptibility factors are frequently nonpathogenic in isolation, and currently identified genetic variants frequently occur in the healthy population to a certain degree. However, in an individual who is immunologically primed, the cumulative loss of an unfortunately high burden of protective factors gives rise to breaks in immune tolerance (indicated by *holes*) that predispose to autoimmunity (eg, dysregulated *IL-2* or *IL-12* signaling pathways) in addition to pathogenic responses to the commensal microbiome (eg, *CARD-9* variants) that result in a clinically identifiable presentation. Additional modifier genes or epigenetic influences may also exist, which influence the rate of progression and variant clinical phenotypes (eg, *Fut-2* polymorphisms).

Despite the evidence of familial aggregation, neither PBC nor PSC display classic Mendelian inheritance. Rather, they exhibit a complex and possibly dynamic gene-gene/gene-environment interaction contributing to disease manifestation at various levels. Therefore, some of the currently proposed genes may influence disease risk by determining how a given individual responds to a particular environmental antigen. Others may act in concert and express the consequence of variation in a stepwise manner and be responsible for diverse clinical phenotypes depending on the coexistence of genetic variability in distinct immune pathways (Fig. 1).

## Human leukocyte antigen associations

The highly polymorphic major histocompatibility complex (MHC) has been implicated in the etiopathogenesis of human autoimmunity for decades, with strong albeit distinct HLA signals recently confirmed for autoimmune liver disease through GWAS.^1^, ^16^ Comprehension of how HLA impacts cholestatic disease mechanistically is somewhat limited, although the fact that an association has been identified in the first instance suggests a defect in the direction and precision of antigen-specific immune responses.

In PBC, several single-nucleotide polymorphisms (SNPs) mapping within or near genes across the HLA region meet the significance threshold for genome-wide association (*P*<5 × 10^−8^), with peak signals mapping between *HLA-DQA1* and *HLA-DQB1*.^17^, ^18^, ^19^, ^20^ PBC-specific associations have also been reported for HLA-DRB1*08, HLA-DRB1*11, HLA-DRB1*14, and HLA-DPB1*03:01, with most corresponding amino acids forming residues in the antigen-binding pocket of the MHC molecule suggesting defective antigen presenting capacity. However, HLA associations vary geographically, with increased PBC susceptibility demonstrated for HLA-DRB1*08:01 in European patients and HLA-DRB1*08:03 – DQB1*06:01 and HLA-DRB1*04:05 – DQB1*04:01 haplotypes implicated in Japanese patients with PBC.^21^ A novel association with the HLA-DRB1*0901 – DQB1*0303 haplotype and progression to cirrhosis and liver transplantation have also been suggested in Japan, whereas HLA-DRB1*13:02 – DQB1*06:04 and HLA-DRB1*11:01 – DQB1*03:01 seem protective.^22^

Pathologically, PBC is characterized by highly conserved humoral and cellular autoreactive immune responses to the mitochondrial pyruvate dehydrogenase complex E2 (PDC-E2).^23^, ^24^ This loss of tolerance has been attributed to the aberrant expression of molecular mimics of PDC-E2 on the cell surface of biliary epithelial cells (BEC), which behave as immunodominant epitopes and bind with *HLA-DRB4*.^25^ However, interactions between other HLA haplotypes and PDC-E2 have not yet determined.

Variation within the MHC region also represents the most significant genetic risk factor for PSC, with proposed SNPs in near-perfect linkage disequilibrium with HLA-B*08:01 as well as more complex associations described for HLA-DRB1*03:01, HLA-DRB1*13:01, HLA-DQA1*01:03, and HLA-DQA1*01:01.^26^, ^27^, ^28^ Simultaneously, strong protective influences of the HLA-DRB1*04 – DQB1*03:02 and HLA-DRB1*07:01 – DQB1*03:03 haplotypes have been documented. Further insight into risk-related alleles in the class-II region of patients has been provided by fine mapping of *HLA-DRB1* genotypes^29^; and 3-dimensional modeling of the corresponding protein chain has identified key amino acids influencing the range of peptides incorporated into the binding pocket of the MHC.

Despite a striking coexistence with colonic inflammation (in ∼80% of cases), most of the HLA associations in PSC are distinct from those identified in IBD, with the exception of a recently identified link to HLA-DRB1*15:01 that is seen to overlap with that of UC (increased risk) and Crohn disease (decreased risk) as well as a multitude of organ-specific autoimmune disease.^30^ The negative prognostic impact of colitis in PSC has been consistently demonstrated in well-characterized patient cohorts and population-based series,^31^, ^32^ with more variable stratification capabilities reported for those patients having elevated serum immunoglobulin G4 (IgG4) levels.^33^, ^34^ Nevertheless, patients with PSC and high serum IgG4 also exhibit an increased frequency of HLA-DRB1*15, the presence of which may, therefore, signify a common high-risk phenotype. Conversely, individuals who manifest the small duct variant of PSC in the absence of concomitant IBD harbor several distinct HLA associations, possibly implying a distinct cholangiopathic entity.^28^, ^35^

## T-cell signaling

In keeping with an immune-mediated cause, PBC and PSC display several immunopathogenic traits common to human autoimmune disease, including overexuberant effector and cytotoxic T-cell responses to pathogen stimulation,^36^, ^37^, ^38^ in parallel to a relative loss of immunoregulatory leukocyte functions.^39^, ^40^

Pathologically, PBC is characterized by a progressive lymphocytic cholangitis centered on smaller intrahepatic bile ducts, and consistent with involvement of the adaptive immune system the infiltrate is predominated by T cells. Large-scale genetic studies have underscored the impact of adaptive regulatory immune pathways; in PBC, this is perhaps best highlighted by interleukin 12 (IL-12) and downstream Janus Kinase (JAK) and Signal Transducer and Activator of Transcription (STAT) signaling.^17^, ^37^, ^41^ IL-12 is central in generating effector type-1 helper T-cell (T_h_1) responses directed toward clearance of intracellular pathogens, and interferon γ (IFNγ) release suppresses IL-23–driven induction of IL-17–producing helper T-lymphocytes (T_h_17).^42^ Additionally, impaired expression of the IL-12 receptor subunit IL-12Rβ2 has been shown to facilitate regulatory T-cell (T_reg_) suppressive functions in the context of a proinflammatory environment. *IL-12A* and *IL-12RB2* variants confer an augmented risk of autoimmunity in many human conditions and have been recently validated in a meta-analysis of several PBC GWAS.^17^, ^18^, ^19^, ^43^, ^44^, ^45^, ^46^ The significance of this observation is elegantly illustrated in experimental cholangiopathy models, wherein mice that lack the p40 subunit of IL-12 (*IL12p40*^−/−^) exhibit dramatic reductions in histologic cholangitis and a significant decrease in the levels of intrahepatic, proinflammatory cytokines.^47^ Many other loci associated with PBC suggest that Toll-like receptor signaling upstream of IL-12 production may also play a role in disease. For instance, IFN regulatory factor-5 interacts with nuclear factor κB (NF*κ*B), which consequently induces expression of several effector T-cell cytokines, including IL-12. Furthermore, variants at the *IL12A* locus have been reported to affect the risk of PBC recurrence following liver transplantation.^48^ Several additional genetic variants involved in key T-cell signaling have been suggested by candidate association studies but not yet emerged as risk loci in PBC GWAS. The classic example here is cytotoxic T-lymphocyte–associated protein-4, which encodes a protein expressed on T-cells and competitively binds to costimulatory molecules CD80 and CD86, thereby ameliorating effector signaling through CD28.^49^

Of interest, *CD28* has emerged as a risk locus in PSC and encodes a T-cell costimulatory molecule necessary for activation and proliferation. A recently published study by Liaskou and colleagues^50^ has demonstrated that in PSC, CD4^+^ T lymphocytes lacking CD28 can be induced by tumor necrosis factor α (TNFα) and infiltrate the peribiliary region where they induce BEC apoptosis through secretion of proinflammatory cytokines in addition to granzyme and perforin-mediated injury. Of note, CD28 is required for IL-2 production, which in turn is required for both the induction (activation of effector T cells) and termination of inflammatory immune responses (induction of T_reg_).

## Tumor necrosis factor α signalling

TNFα is an activating factor for several intracellular pathways that determine the fate of epithelial cells, including hepatocytes and BEC.^51^ Interactions between specific members of the TNF pathway lead to the induction of apoptosis as well as activation of NF*κ*B signaling; and in PBC, GWAS have identified 3 loci containing genes in TNFα signaling pathways.^18^, ^20^, ^52^ Macrophages from patients with PBC when stimulated with apoptotic bodies from BEC produce high levels of TNFα, with serum levels of TNFα reflecting the severity of intrahepatic damage.^23^, ^53^

A prominent role for TNFα in the immunopathogenesis of PSC has also been suggested through induction of immunopathogenic T-cell phenotypes^50^ as well as indirectly through the hepatic endothelial induction of mucosal chemokines and adhesion molecules that are normally gut restricted in an NF*κ*B-dependent manner.^54^ Moreover, PSC genetic risk associations include the 1p36 locus that encompasses the gene encoding TNF-superfamily receptor TNFRSF14, a protein expressed on CD4^+^ and CD8^+^ T cells, B cells, monocytes, neutrophils, dendritic cells, and mucosal epithelium, which behaves as a molecular switch modulating lymphocyte activation.^55^

## Mucosal immune activation in liver autoimmunity

T_h_17 cells are abundant in the intestinal lamina propria where they are induced by commensal bacteria and provide protection against invading pathogens.^56^, ^57^ In mice, peripheral T_h_17-cells can be redirected from the periphery to the small intestine via chemokine recruitment through CCR6-CCL20 interactions; and in humans, CCL20 is expressed on inflamed bile ducts, suggesting that the same chemokine pathway might promote accumulation in the inflamed liver.^58^ Of interest, the recent PBC GWAS meta-analysis by Cordell and colleagues^46^ identified *CCL20* as a plausible candidate gene, which, given the role of this chemokine axis in the formation and function of gut lymphoid tissues, suggests a pivotal role of the mucosal immune system in the initiation or perpetuation of lymphocytic cholangitis.^59^

The chemokine receptor CXCR5 has also been identified as a risk locus in PBC^18^ and is involved in the migration of both T lymphocytes and B lymphocytes to sites of antibody production along a chemokine gradient (ligand CXCL13). CXCR5 is constitutively expressed on mature B lymphocytes and induced on T-follicular helper cells (T_Fh_) in response to antigen and is critical to formation of intestinal lymphoid follicles.^60^ Emerging evidence also indicates that CXCR5 deficiency is associated with defective germinal center responses within the liver, the critical location for driving B-lymphocyte differentiation.^61^ This observation is of particular interest given that patients with PBC exhibit an increased frequency of T_FH_ cells in vivo that correlates with increased B-cell activation, disease severity, and biochemical response to ursodeoxycholic acid.^61^ IL-7 is another key player for both T and B lymphocyte development and is also necessary for sustaining peripheral T-cell populations. Receptor induction occurs on T-cell positive selection in the thymus and directs thymic CD8^+^ lineage specification and peripheral naïve T-cell homeostasis, whilst simultaneously having a role in myeloid cell differentiation.^62^, ^63^ IL-7R expression is generally reduced on T_reg_ compared with other T-cell subsets, and IL-7 signaling plays an important role in the imprinting of a gut-tropic (α4β7-integrin positive) phenotype^64^ —a noteworthy observation given that mucosal lymphocytes purportedly drive proinflammatory responses in autoimmune cholestasis.^59^, ^65^

**Fig. 2:**
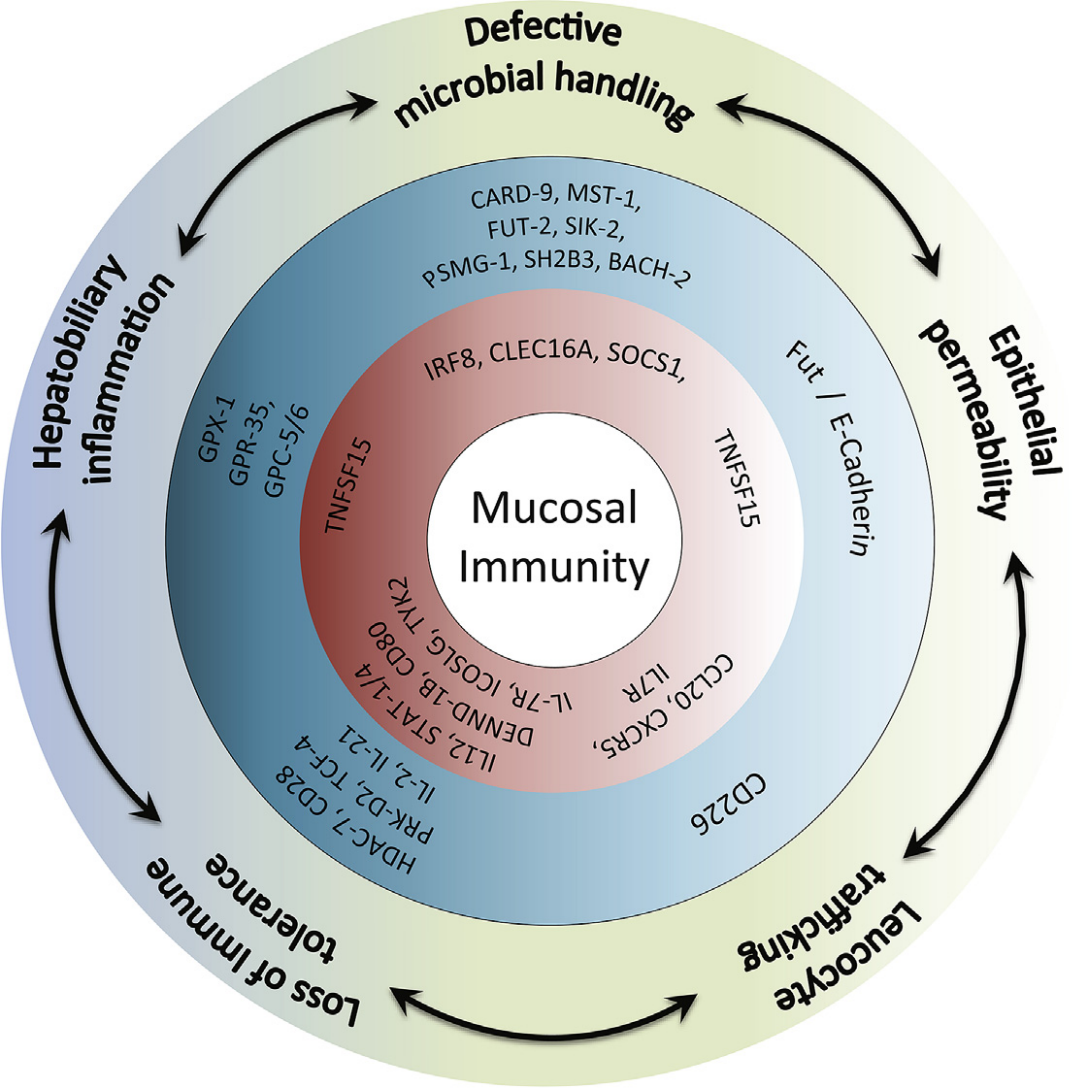
Mucosal genetics in autoimmune cholestatic liver disease. The strongest genetic associations in autoimmune cholestatic liver disease are within the MHC. However, a significant proportion of non-HLA associations and epigenetic influences underscore the importance of mucosal immunogenicity in the pathogenesis of autoimmune cholangitis. These associations include defective microbial handling and immunopathogenic responses to the commensal microbiome, defects in epithelial (eg, intestinal or biliary) barrier function, dysregulated leukocyte trafficking and homing to sites of injury, loss of intestinal and hepatobiliary tolerogenic responses, and consequently direct tissue inflammation. The outer (*green*) ring in this diagram indicates the putative mucosal pathway, with PSC risk genes identified by the middle (*blue*) ring and PBC risk genes the inner (*red*) ring.

Genetic links to mucosal immunity are even more evident in PSC (Fig. 2).^59^ The importance *IL-2/IL-2Rα* polymorphisms, suggested through associations at the 4q27 and 10p15 loci, respectively,^26^ is supported by the fact that mice lacking IL-2Rα develop autoantibodies and a T-cell–mediated cholangitis together with colitis.^66^ Moreover, liver-derived lymphocytes from patients with PSC show reduced expression of the IL-2 receptor and an impaired proliferative response to pathogen stimulation *in vitro*.^67^ IL-2 can contribute to termination of inflammatory immune responses by promoting the development, survival, and function of T_reg_. Loss of IL2Rα signaling function in PSC is supported by the observation that patients who harbor variant polymorphisms exhibit reduced circulating populations of T_reg_.^39^

An immunosuppressive role for histone deacetylase *(HDAC)-7*, a gene implicated in the negative selection of T cells in the thymus and development of tolerogenic immune responses,^26^ is supported by a genetic association at 12q13 in PSC GWAS in which the most associated polymorphism was located within an intron encoding serine-threonine protein kinase *(PRK)-D2* (19q13). When T-cell receptors of thymocytes are engaged, PRKD2 phosphorylates HDAC7 resulting in loss of its gene regulatory functions. This gives rise to apoptosis and negative selection of immature T cells. Notably, this negative selection takes place owing to a loss of HDAC7-mediated repression of the leukocyte transcription factor *Nur77*.^26^ *Nur77* expression parallels that of *IL-10* and is heavily influenced by salt-inducible kinase *(SIK)-2* polymorphisms, the latter of which is also proposed as a genetic risk-locus in PSC. Of note, *IL-10* variants are an established susceptibility factor for early onset ulcerative colitis,^68^ and exposing *Il10*^−/−^ mice to a diet high in saturated fat has been shown to induce specific changes in the bile acid pool that consequently leads to alterations in the gut microbiome and increased susceptibility to IBD^69^ – linking multiple putative risk loci to a common mucosal pathway in PSC. Further impression of impaired mucosal tolerance is suggested through a genetic association at 18q21, which contains transcription factor-4; congenital deficiency of which not only results in partial blockade of early B- and T-cell development but also attenuated development of plasmacytoid dendritic cells (pDC) in murine models.^70^

Caspase-recruitment domain (CARD)-9 is an important downstream mediator of signaling from mucosal pattern-recognition receptors (PRR), and genetic associations suggest a link between defective intestinal mucosal microbial handling and the development of PSC.^71^ *Card9*^–/–^ mice seem more susceptible to experimentally-induced colitis and typified by defective IFNγ and T_h_17 responses, as well as reduced transcription of the mucosal chemokine CCL20; signifying the critical importance of CARD9 in the maintenance of epithelial immunostasis.^72^ Another one of the strongest non-HLA associations in PSC is macrophage-stimulating (*MST*)*-1*, which is also associated with UC and Crohn disease. MST-1 is expressed by BEC and involved in regulating innate immune responses to bacterial ligands, as well as modulating lymphocyte trafficking in lymphoid tissues through integrin- and selectin-mediated adhesion.^73^, ^74^, ^75^ Glutathione peroxidase (GPX)-1 is an antioxidant enzyme located close to MST-1, and polymorphisms in *GPX-1* may also confer an increased disease susceptibility to PSC.^29^ Moreover, *Gpx1/2*^−/−^ mice develop a chronic ileocolitis with an increased frequency of colonic malignancy.^76^

Variants in *Fut-2*, an enzyme encoding galactoside 2-alpha-l-fucosyltransferase-2, have also been suggested to confer increased susceptibility to PSC (as well as Crohn's disease), although fall short of reaching significance at a genome-wide level.^77^, ^78^ Fucosyltransferase variants alter the recognition and binding of various pathogens to carbohydrate receptors on the mucosal surface and are associated with changes in the commensal phyla in affected patients with PSC characterized by elevated *Firmicutes* and reduced *Proteobacteria*. These aforementioned microbial changes are akin to that observed in *FUT-2* mutations associated with Crohn's colitis and again links defective immune responses to the gut microbiota in PSC. Moreover, variants in *FUT-2* have been described as a risk factor for the development of dominant biliary stenosis in PSC, a putative surrogate of adverse clinical outcomes.^34^, ^79^

An increased lifetime risk of hepatobiliary carcinoma as well as colorectal malignancy is well recognized in PSC^32^ and previous studies have indicated that the latter is associated with altered fucosylation of the adhesion molecule E-cadherin.^80^ A recent study in mice has illustrated that congenital E-cadherin deletion results in spontaneous periportal inflammation and periductal fibrosis, in addition to an enhanced susceptibility to hepatobiliary cancer, akin to clinical PSC, implying that cholangitis and oncogenesis are a direct result of defective pathogen sensing.^81^

## Immuno-epigenetic influences

Less than 20% of the heritability of autoimmune cholestatic liver diseases have been uncovered by GWAS, and it is likely that some of the missing risk is attributable to environmental triggers or nonhereditary genetic influences. As a female preponderant disease, the frequency of preferential X-chromosome monosomy on peripheral lymphocytes seems to increase with age, at a rate significantly greater compared with normal and non-PBC liver disease-matched controls.^82^, ^83^ Of further interest is the increased rate of Y-chromosome loss in men with PBC,^84^ suggesting that X-linked alleles or haplotypes predispose to autoimmunity as a result of haploinsufficiency irrespective of sex.

Support of this hypothesis has recently been provided by the Milan PBC Epigenetic Study Group who report striking demethylation of the *CXCR3* promoter that inversely correlated with receptor expression in peripheral blood CD4^+^ T cells.^85^ This finding is of particular significance given that CXCR3 is highly expressed on T_h_1 and T_h_17 liver-infiltrating CD4^+^ cells, and the cognate ligands (CXCL9–11) are known to be upregulated on the damaged bile ducts in PBC liver.^86^ A further epigenetic observation is reduced methylation of the CD40-ligand promoter regions among patients with PBC compared with controls,^87^ which is of particular interest given the importance of CD40 in T- and B-cell interactions. Of note, elevated circulating levels of CD40 have been detected in the serum of patients with systemic autoimmune diseases^88^ and ectopic B-cell expression reportedly associated with intestinal inflammation.^89^

## Therapeutic considerations and future outlook

The combined output from GWAS and associated works thus far provides explanation for less than 20% of disease heritability in PBC and PSC.^1^ Therefore, clinical merits of genomic studies will only be fully realized when genetic and epigenetic data can link to the gut microbiome and environmental influences that collectively occupy the complex orchestra of disease pathogenesis, akin to that which has been described for celiac disease.^90^

Simultaneously, a stratified approach to therapy is hoped to arise that focuses on carefully selected patient populations and structured care delivery.^34^ For instance, specific transcriptional signatures enriched for genes involved in memory T-cell generation and receptor-signaling (including IL-7) have been described in UC and Crohn's disease that accurately predict colectomy risk from the point of diagnosis; it is plausible that such bioindicators also exist in immune-mediated liver diseases given the overlapping defects in mucosal immunogenicity.^91^ A further, major aim of genetic studies in PBC and PSC has been in the identification of ostensible avenues for future therapeutic exploration. The wealth of overlapping susceptibility loci that are shared with other autoimmune diseases has been extensively discussed in several recent articles,^1^, ^3^, ^4^, ^92^ which collectively imply a common genetic architecture underlying immune-mediated tissue injury. This hypothesis needs to be tested and confirmed but, if correct, suggests novel approaches to treatment in which regulatory pathways are enhanced or effector responses suppressed by preventing the activation and recruitment of immunopathogenic cell populations (Table 1).

Presently, there is a large shortfall between the available genetic information to date and permeation into clinical practice, a providence that PBC and PSC share with other complex diseases studied on a genome-wide scale. Nevertheless, the advances to date in understanding genetics of chronic cholestasis speak broadly to the ultimate goal of all such studies: to guide treatment that is biologically driven and mechanistically linked.

## Figures and Tables

**Table 1:** Conceivable therapeutic targets arising from genetic and epigenetic studies

Pathway	Intervention and Rationale	Expedients	Precedents
IL-12/IL-23	*PBC* IL-12 drives differentiation of activated, naïve T-cells to IFNγ-producing T_h_1 cells, contributing to loss of tolerance in several models of autoimmunity. Murine models of cholangiopathy also exhibit a milder hepatobiliary phenotype in the absence of functional IL-12. IL-23 (which shares a common p40 subunit with IL-12) is also essential for differentiation of T_h_17 responses, CD8-mediated IL-17 release and implicated in the breakdown of immune self-tolerance.	Anti-IL-12/23 (ustekinumab) Anti-IL-17A (secukinumab/ixekizumab) Anti-IL17RA (brodalumab)	Crohn disease^93^ Psoriasis Psoriasis^94^ Uveitis Ankylosing spondylitis Crohn disease^94^
NF*κ*B	*PBC* and *PSC* Nuclear transcription factor with pleiotropic effects, including regulation of expression of human endothelial adhesion molecules responsible for leukocyte recruitment (eg, VAP-1 and MAdCAM-1), as well as pathways involved in T-cell activation (eg, CD80/CD86).	Anti-CD80 (abatacept) Anti-α4β7 - cognate integrin for MAdCAM-1 (vedolizumab)	Intestinal inflammation^95^, ^96^
CD40–CD40L	*PBC* CD40–CD40L interactions are critical for T-cell–B-cell interactions and elevated circulating CD40 levels recognized in a host of human autoimmune diseases. CD40 antagonists have been shown to be effective in inducing remission from experimentally induced colitis, hematological malignancies and autoimmune encephalitis.	Anti-CD40 (dacetuzumab/lucatumumab)	Multiple sclerosis^97^ (preclinical) Chronic lymphatic leukemia, non-Hodgkin lymphoma, multiple myeloma^98^
CXCR3–CXCL9/10/11	*PBC* CXCR3 expression is upregulated on liver-infiltrating T_h_1 and T_h_17 cells in early stage PBC and the corresponding ligands secreted in larger quantities by inflamed (versus noninflamed) BEC.	Anti-CXCL10 (MDX-1100)	Rheumatoid arthritis^99^
CXCR5–CXCL13	*PBC* This chemokine axis guides both B- and T-cell positioning along CXCL13 chemokine gradients and facilitates migration to germinal centers.	Anti-CXCL13 (MAb 5261)	(Preclinical development)^100^
CCL20–CCR6	*PBC* Responsible for the recruitment and positioning of T-cells (predominantly T_h_17 cells) around inflamed BEC.	Anti-CCR6	(Preclinical development)
ORMDL3	*PBC* Represents one of several putative risk genes at the 17q12–21 locus and regulates eosinophil trafficking and coexpression of α4 integrins. ORMDL3 is also observed to predict response to corticosteroids in childhood asthma.^101^, ^102^	May help to identify corticosteroid response in selected patients	-
GPR35	*PSC* Expressed by intestinal epithelial cells in the intestine and in multiple leukocyte subtypes. Specific activation of GPR35 has been demonstrated to significantly reduce IL-4 release from natural killer T cells. PRKD2 polymorphisms are associated with early onset IBD.^103^, ^104^	Anti-GPR35	Antibody recently developed; clinical applications not yet specified^105^
PRKD2/HDAC7/Nur77/SIK2	*PSC* A serine-threonine protein kinase, which phosphorylates HDAC7; this gives rise to nuclear exclusion and loss of gene regulatory functions, ultimately resulting in apoptosis and negative selection of immature T cells due to a loss of HDAC7-mediated repression of Nur77, which is regulated by SIK2.	Anti-PRKD2	(Preclinical development)^106^
